# ‘SGoFicance Trace’: Assessing Significance in High Dimensional Testing Problems

**DOI:** 10.1371/journal.pone.0015930

**Published:** 2010-12-29

**Authors:** Jacobo de Uña-Alvarez, Antonio Carvajal-Rodriguez

**Affiliations:** 1 Departamento de Estadística e Investigación Operativa, Facultad de Ciencias Económicas y Empresariales, Universidad de Vigo, Vigo, Spain; 2 Departamento de Bioquímica, Genética e Inmunología, Facultad de Biología, Universidad de Vigo, Vigo, Spain; Telethon Institute of Genetics and Medicine, Fondazione Telethon, Italy

## Abstract

Recently, an exact binomial test called SGoF (Sequential Goodness-of-Fit) has been introduced as a new method for handling high dimensional testing problems. SGoF looks for statistical significance when comparing the amount of null hypotheses individually rejected at level γ = 0.05 with the expected amount under the intersection null, and then proceeds to declare a number of effects accordingly. SGoF detects an increasing proportion of true effects with the number of tests, unlike other methods for which the opposite is true. It is worth mentioning that the choice γ = 0.05 is not essential to the SGoF procedure, and more power may be reached at other values of γ depending on the situation. In this paper we enhance the possibilities of SGoF by letting the γ vary on the whole interval (0,1). In this way, we introduce the ‘SGoFicance Trace’ (from SGoF's significance trace), a graphical complement to SGoF which can help to make decisions in multiple-testing problems. A script has been written for the computation in R of the SGoFicance Trace. This script is available from the web site http://webs.uvigo.es/acraaj/SGoFicance.htm.

## Introduction

Multiple-testing problems have received much attention since the advent of the ‘-omic’ technologies: genomics, transcriptomics, proteomics, etc. They usually involve the simultaneous testing of thousands of hypotheses, or nulls, producing as a result a number of significant *p*-values or effects (that is, an increase in gene expression, or RNA/protein levels). An important issue here is the control of type-I errors (false positives). In this regard, there are several multiple-testing algorithms that focus on controlling the family-wise error rate (FWER), defined as the probability of committing at least one type-I error through the several hypotheses tested. Some of these methods control the FWER in a strong sense (i.e. under all configurations of the true and false hypotheses), while others only require a weak control of the type-I error. Weak control means that the FWER is maintained below a given error level under the intersection (or complete) null hypothesis. Unfortunately, past research showed that methods controlling the FWER have a remarkable lack of power, that is, the proportion of false negatives is too large. To overcome this drawback, the false discovery rate (FDR) has been introduced as a less stringent criterion leading to more powerful procedures. The FDR is defined as the expected proportion of rejected hypotheses that are false positives. Traditional FWER- and FDR- based methods are nicely reviewed by Nichols and Hayasaka [1] as well as by Dudoit and Laan [2]. However, the power (i.e. the proportion of true positives among the rejected hypotheses) of the FDR-based methods decreases with the number of tests, being unable to detect even one effect in particular situations [3]. The *q*-value, introduced by Storey and Tibshirani [4] as an extension of the FDR criterion, is a possible solution to this problem. The *q*-value reports the FDR associated to each rejection threshold for the sequence of *p*-values. In this way, after a preliminary analysis, the researcher may choose the FDR level depending on the number of effects he/she wants to find, or the maximum acceptable threshold for the *p*-values. Still, this approach does not provide an objective answer to two important questions:

1. How many effects should be declared?
2. Which FDR is reasonable to assume for the discoveries in a given experimental setup?

Recently, Carvajal-Rodríguez et al. [3] introduced a new method (SGoF) for handling the simultaneous testing of *S* hypotheses. The basic idea under SGoF is to compare the number of hypotheses individually rejected at level γ = 0.05 with the number expected under the intersection null, S*γ, looking for statistical significance in that comparison. The alternative hypothesis is that the expected number of rejected nulls at γ level is above S*γ, so a one-sided test is performed. More explicitly, let *p*_1_<*p*_2_<…<*p*_S_ to be the sorted *p*-values associated to the *S* nulls. Then, the observed number of individual rejections at γ level is *K*(γ) = #{*p*_i_≤γ }. Under the intersection null, *K*(γ) follows a Binomial distribution with parameters *S* and γ; the *p*-value of *K*(γ) is computed from such distribution, and the decision of ‘accepting/rejecting the intersection null’ is taken accordingly. More explicitly, if α = 0.05 is the significance level for such a test, the intersection null is rejected whenever *K*(γ)≥*b*_α_(γ), where *b*_α_(γ) is the 100(1-α)% percentile of the Binomial(*S*, γ) distribution. This guarantees that the FWER is controlled at level α in the weak sense [3]. In case of rejection, SGoF declares as true effects the *K*(γ)−*b*_α_(γ)+1 smallest *p*-values. This is intuitive because the associated tests are responsible for the decision of rejecting the intersection null of no effect.

The SGoF can be interpreted/described as a sequential algorithm that in a step-wise mode decides if a candidate *p*-value corresponds or not to a true effect. In the first step, the algorithm compares *K*(γ) to S*γ, as discussed above; if the intersection null is rejected, the value of *K*(γ) is updated to be *K*(γ) - 1, and the process is repeated until significance is lost. In this way, SGoF performs a systematic test for proportions, which provides the significance associated to each *p*-value. The SGoF method (Sequential Goodness-of-Fit) takes its name from this iterative algorithm, which is described in complete detail in Carvajal-Rodríguez et al. [3].

One of the main advantages of SGoF is that it exhibits an increasing power with the number of tests. As mentioned, this is not true for other multiple-testing corrections (including those controlling the FDR rather than the FWER), which, under some settings, can hardly find even one true effect in high dimensions [3]. The key for this desirable property of SGoF is that the method concentrates on the discrepancy between the observed and expected numbers of *p*-values below a given threshold, without regard to any *a priori* proportion of false discoveries. Since the number of true effects declared by SGoF (*K*(γ)−*b*_α_(γ)+1) leads to an estimated FDR, the new method informs in an indirect way about ‘which FDR is reasonable to assume’ for a given data set, answering the former questions (1) and (2) at the same time. As stated before, this information is not provided by other existing methods, for which the choice of the FDR parameter must be subjective.

The original implementation of SGoF concentrates in the case γ = α ( = 0.05). Obviously, the choice γ = α, while being intuitive, is not essential for the SGoF procedure. Therefore, it could be interesting to look at SGoF results when γ moves away from α. The only caution that should be taken is that *p*-values above α can be declared as true effects if γ≠α (see the Real Data Illustration section). Interestingly, we have confirmed through simulations (see Simulations section) and real data analysis (see Real Data Illustration section) that, as expected, the SGoF power may be increased if different choices for γ are taken into account. This idea leads us to consider a significance trace of SGoF, which is basically the level of significance of SGoF (and some associated measures of performance and summary results) when the γ parameter varies on the interval (0,1) in a continuous way. This ‘SGoFicance Trace’ (from SGoF's significance trace) is introduced in the corresponding section. Alternatively, there is the possibility of choosing a single γ value for SGoF, based on some optimality criterion. This line is explored in an independent paper (Carvajal-Rodríguez and Uña-Alvarez, in preparation).

## Results and Discussion

### Simulations

We have simulated the simultaneous testing of *S* = 1,000 null hypotheses among which 100 are false (an effect of 10%). The *p*-values corresponding to the true nulls were simulated from a uniform distribution. The *p*-value of each effect was randomly drawn as *p* = 1−Φ(*Z*+*w*), where *Z* is a standard normal deviate, Φ is the cumulative distribution function of the standard normal, and *w* is a real number representing the effect level. If we think about a one-sided normal test for the mean, *w* can be identified as the distance between the null and the alternative mean values, normalized by the sample standard error of the mean. In the simulations we took *w* = 2.

**Figure 1:**
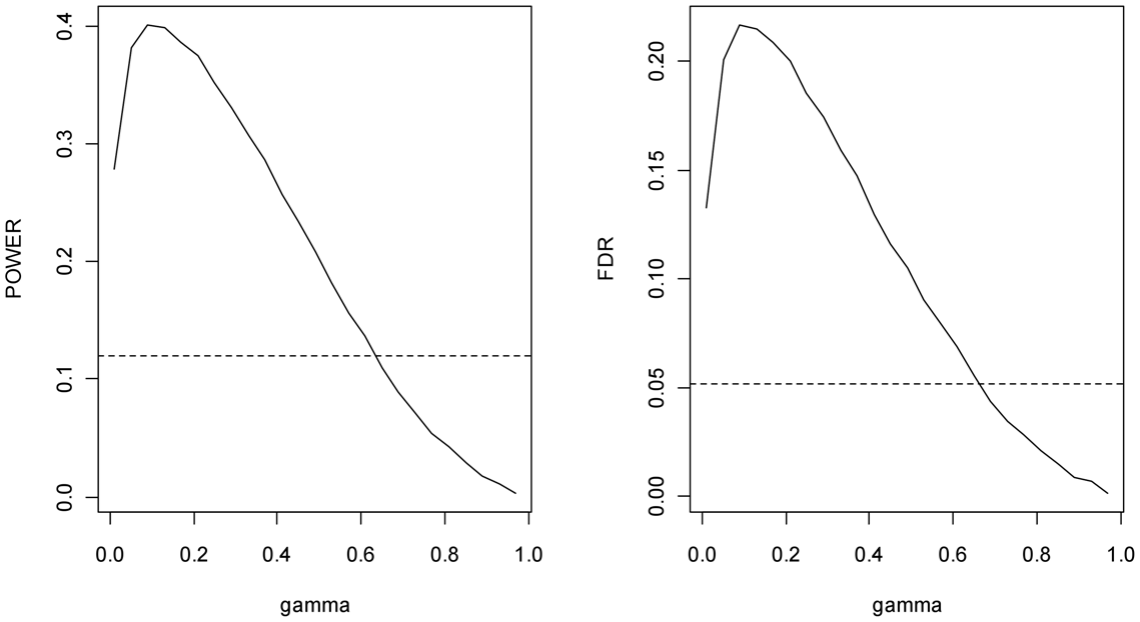
Power (left) and FDR (right) of SGoF depending of γ. The dashed lines correspond to the BH method at nominal FDR of 0.05. The number of hypotheses is 1,000 and there is an effect of 10%. Averages were computed through 1,000 Monte Carlo trials.

In Figure 1 we give the power of SGoF (defined as the proportion of effects detected among the 100 existing ones) as well as the FDR for a grid of γ values ranging from 0.01 to 0.99. Weak control of FWER (α) was taken as 0.05. The results correspond to averages through 1,000 Monte Carlo trials. For comparison purposes, the power (12.01%) and the FDR (5.16%) attained through the simulations by the Benjamini-Hochberg (BH) method [5] (with nominal FDR of 0.05) are also provided (dashed line). These results illustrate that (a) SGoF was able to detect many more effects than BH (in accordance with [3]); and (b) the power of SGoF was greatly influenced by the γ parameter, being maximized around γ = 0.09. Moreover, the trace of the SGoF power indicate that a ‘reasonable FDR to pay’ in this setup would be no more than 22%, a conclusion that cannot be reached neither from the BH method nor from other usual multiple-testing corrections. Hence, there is a clear motivation for this type of plots in real data applications, not focusing on any *a priori* choice of γ. This is what the SGoFicance Trace provides.

Other choices of *w* and of the percentage of true effects provide similar evidences. As a summary, in Table 1 we provide the power and FDR attained by SGoF(γ) for γ = 0.01, 0.05, 0.09, 0.13 and 0.17 for several percentages of effects among the 1,000 hypotheses being tested (10% and 30%), with different degree of departure from the null hypothesis (*w* = 1, 1.5 and 2). The numbers reported are averages along 1,000 Monte Carlo simulations from the model. Again, results corresponding to the BH method (at nominal FDR of 5%) are provided for comparison. From Table 1 it is seen how the power of SGoF's method can be increased by changing the value of the γ parameter. It is also seen that the FDR control of the BH method may be too strict in the sense of power; for example, for a 30% of true effects with a weak deviation from the null (*w* = 1), BH is only able to detect a 0.7% of the non-true nulls (the power of SGoF is one order of magnitude greater in this case). This agrees with previous results on the possible lack of power of BH [3].

**Table 1:** Average power and FDR of SGoF (α = 0.05) depending on γ.

		SGoF(0.01)	SGoF(0.05)	SGoF(0.09)	SGoF(0.13)	SGoF(0.17)	BH
10%*w* = 2	Power	0.2786	0.3815	0.4004	0.3984	0.3857	0.1202
	FDR	0.1327	0.2004	0.2164	0.2145	0.2086	0.0516
10%*w* = 1.5	Power	0.1202	0.1958	0.2198	0.2237	0.2242	0.0211
	FDR	0.2160	0.2963	0.3203	0.3224	0.3228	0.0503
10%*w* = 1	Power	0.0259	0.0529	0.0691	0.0757	0.0801	0.0028
	FDR	0.2932	0.3779	0.4087	0.4140	0.4222	0.0472
30%*w* = 2	Power	0.3314	0.4998	0.5402	0.5499	0.5481	0.3340
	FDR	0.0504	0.0977	0.1117	0.1157	0.1152	0.0507
30%*w* = 1.5	Power	0.1642	0.3042	0.3556	0.3767	0.3871	0.0824
	FDR	0.0878	0.1444	0.1682	0.1785	0.1830	0.0496
30%*w* = 1	Power	0.0568	0.1312	0.1688	0.1887	0.2019	0.0067
	FDR	0.1683	0.2323	0.2583	0.2713	0.2795	0.0505

The number of hypotheses is 1,000 and the proportion of non-true nulls is 10% or 30%. Strong (*w* = 2), intermediate (*w* = 1.5) and weak (*w* = 1) effects are considered. BH method at FDR of 5% is included. Averages were computed through 1,000 Monte Carlo trials.

### The SGoFicance Trace

**Figure 2:**
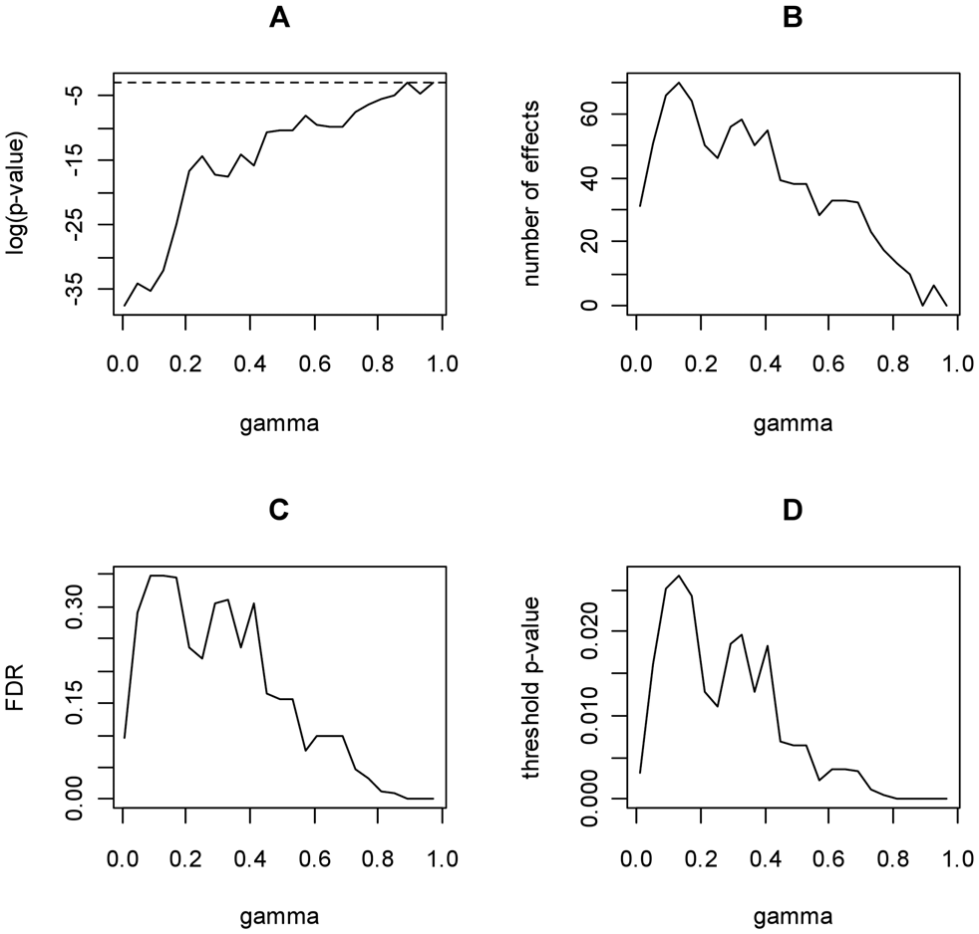
SGoFicance Trace at FWER 5% for the Monte Carlo trial #101 in the simulation study. (A): SGoF's log-significance plot; (B): SGoF's number of effects; (C): SGoF's FDR; (D): SGoF's threshold for *p*-values. The number of hypotheses is 1,000 and there is an effect of 10%. The proportion of true nulls was taken as its true value (0.9) for the computation of FDR's.

The SGoFicance Trace is a graphical device constructed from the SGoF multitest. Let SGoF(γ) denote the SGoF algorithm described in the Introduction when using a given threshold γ. The basic idea is to let γ vary on the whole interval (0,1). The SGoFicance Trace displays up to four different plots: (A) log-significance (*p*-value) of SGoF(γ) vs. γ; (B) number of effects detected by SGoF(γ) vs. γ; (C) estimated FDR of SGoF(γ) vs. γ; (D) threshold of the *p*_i_'s vs. γ. Figure 2 reports the SGoFicance Trace pertaining to one of the Monte Carlo trials in the previous section (see below). The definition of each plot and the information it provides is discussed below.

#### SGoF's log-significance plot

The first plot (Figure 2A) displays the *p*-value (in log-scale) of SGoF(γ) for each value of γ. Namely, the displayed values were computed as the probability tail of the Binomial(*S*, γ) distribution to the right of *K*(γ), that is *p*(γ) = *P*(Binomial(*S*, γ)≥*K*(γ) ). The log-scale is used because the *p*-values are expected to be small along most of the γ-grid, with an exponential increment as γ approaches to one. This plot reveals the amount of significance contained in SGoF(γ) against the intersection null, for each γ value. Since the *p*_i_'s pertaining to the non-true nulls would tend to be located close to zero, a monotone increasing shape is expected in this plot. An horizontal dashed line at point log(α) was incorporated to the plot for completeness. Here, α represents the FWER that is controlled by SGoF(γ) in a weak sense. The default value for α was set at 0.05.

#### SGoF's number of effects

For each γ, the number of effects declared by SGoF(γ) at FWER α is *N*_α_(γ) = *K*(γ)−*b*_α_(γ)+1. Unlike *p*(γ), this number depends on both α and γ. The second plot (Figure 2B) in the SGoFicance Trace displays the *N*_α_(γ) values against γ, for the particular choice α = 0.05. In a typical multiple-testing problem, an inverted U-shape will be found in this plot, meaning that the largest number of effects corresponds to intermediate values of the γ parameter. Notice that, while *K*(γ) is an increasing function of γ, *b*_α_(γ) also increases as γ gets larger.

#### SGoF's FDR

A commonly used measure of performance in multiple-testing problems is the FDR. Hence, it is interesting to evaluate the FDR of SGoF(γ) for each value of γ. In practice, the FDR is unknown, but some estimation methods can be used to find it. The FDR estimation procedure starts from some preliminary assessment of the proportion of true nulls, π_0_. Different methods have been proposed in the literature to do so. We have implemented the method proposed by Dalmasso et al. [6] with *n* = 1 (see equation (6) in [6]) because of its simplicity and reasonable behavior. However, other estimation methods are possible [7], [8]. Explicitly, we estimate π_0_ by the average of the -log(1 - *p*_i_)'s. Denoting this quantity by *e*π_0_, the estimated FDR of SGoF(γ) was just eFDR_α_(γ) = S*q_α_(γ)*eπ_0_/*N*_α_(γ), where *q*_α_(γ) stands for the threshold of the *p*i's needed to reach *N*_α_(γ) effects, that is, *q*_α_(γ) is such that *N*_α_(γ) = #{*p*_i_≤*q*_α_(γ)}.

As for the plot of the SGoF's number of effects, the FDR plot (Figure 2C) will typically show a concave form, with a maximum around the point at which *N*_α_(γ) attains its largest value. Recall that, unlike the FDR-based methods, SGoF is not constructed to respect a given fixed proportion of false discoveries. For this reason, this plot is informative about the ‘reasonable amount of FDR’ that could be faced in a given situation. Again, the nominal FWER α was set to 0.05.

#### SGoF's threshold for p-values

Finally, the SGoFicance Trace provides a plot showing the threshold values *q*_α_(γ) *versus* γ, in the case α = 0.05. Interestingly, in this way the significance level that results after the application of SGoF(γ) for each individual null hypothesis can be investigated.

To resume the above explanations, the SGoFicance Trace at a FWER of 5% corresponding to the randomly chosen Monte Carlo trial #101 (from the simulation study described in the first section) is shown in Figure 2. In this case, we see that SGoF was able to detect an effect, i.e. *p*<0.05, independently of the γ value (Figure 2A). In addition, the highest number of effects (70) was detected by SGoF based on γ = 0.13 (Figure 2B). The SGoF's FDR plot (Figure 2C) indicated that this maximum detection led to a false discovery proportion of about 35%. Moreover, it also tells us that the researcher should never assume more than that rate for that particular situation. In other words, under SGoF's view there are no more than 70 *p*-values that could be declared as true effects in a ‘reasonable way’. The threshold *q*_α_(γ = 0.13) such as that 70 = #{*p*_i_≤*q*_α_(γ)} (Figure 2D) was 0.0267, but according to the Figure it will be different if the γ parameter of SGoF changes.

### Real data illustration

As an illustrative example, we took the microarray study of hereditary breast cancer by Hedenfalk et al. [9]. One of the goals of this study was to find genes differentially expressed between BRCA1- and BRCA2-mutation positive tumors. Thus, for each of the 3,226 genes of interest, a *p*-value was assigned based on a suitable statisticical test for the comparison. Following previous analysis of these data [4], 56 genes were eliminated because they had one or more measurements exceeding 20. This left *S* = 3,170 genes. According to the method proposed by Dalmasso et al. [6] with *n* = 1, there is a proportion of true nulls of 71.77%, i.e. about 895 true effects. On the basis of the *q*-value method, Storey and Tibshirani [4] found 160 genes with significant differential expression at 5% of FDR (that is, about 8 genes are expected to be false positives). The amount of significant effects may be increased up to above 300 by letting the FDR rise up to 10% [4]. However, what the *q*-value method does not tell us is which FDR should be reasonably assumed for the Hedenfalk et al. [9] data.

**Figure 3:**
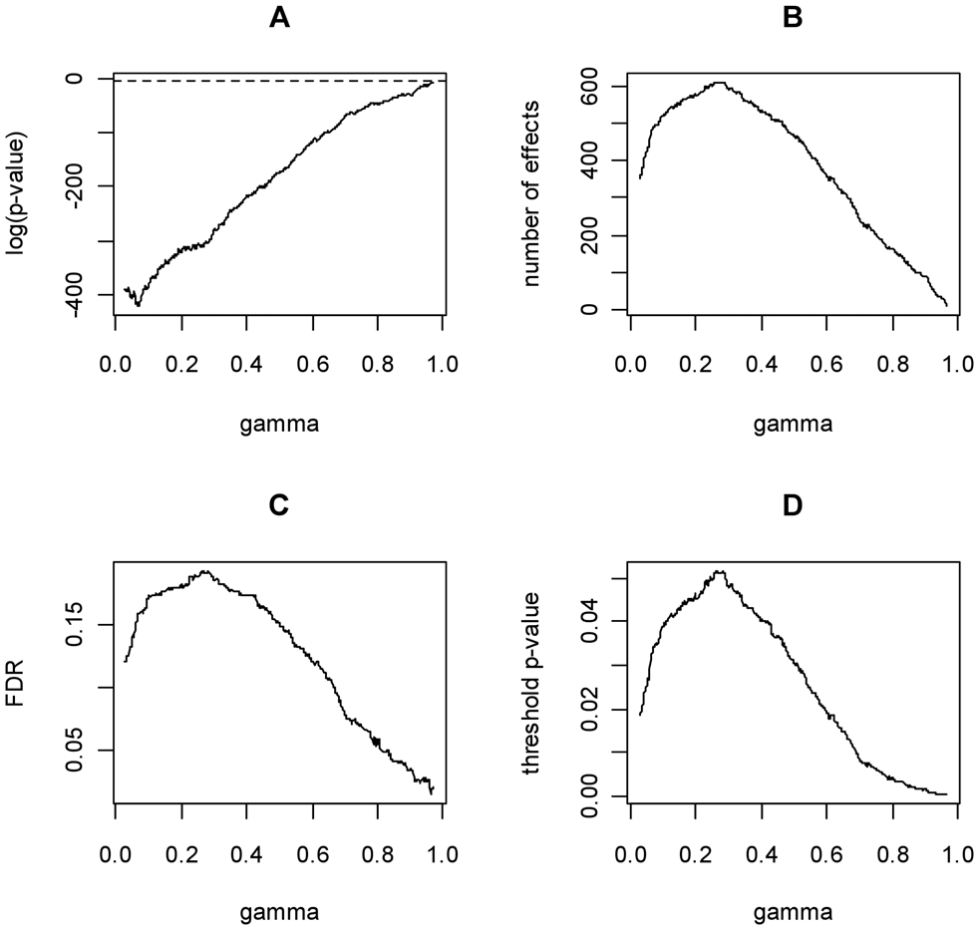
SGoFicance Trace at FWER 5% for the 3,170 genes of Hedenfalk et al. (2001). (A): SGoF's log-significance plot; (B): SGoF's number of effects; (C): SGoF's FDR; (D): SGoF's threshold for *p*-values. The proportion of true nulls for the computation of the FDRs was estimated according to Dalmasso et al. [6], case *n* = 1.

We have computed the SGoFicance Trace at a FWER of 5% for this data set and the result is displayed in Figure 3. The first relevant finding is that SGoF was able to detect up to 613 effects by committing an FDR of 20%, so, there is no reason to assume a higher FDR. These results correspond to the application of SGoF(γ) with γ = 0.26, meaning that, among the 1473 *p*-values below 0.26, there is statistical evidence that at least 613 correspond to non-true nulls. One could argue that the q-value method would also be able to declare the same amount of effects just by raising the FDR up to 20%. This is true, but it is only possible as a posteriori analysis. In addition with the q-value method there is no hint to choose a specific FDR value. For example the researcher could decide to assume 15, 20 or still 25% FDR. However SGoF automatically tells that (under its view) there is no statistical significance beyond the detected 613 effects and therefore gives the corresponding FDR. So, at the end, one reaches the conclusion that 613 effects can be declared at maximum and that, in this case, about 123 of them will correspond to false positives. There is no reason to assume a higher FDR since this will not increase the number of effects detected by SGoF. A second relevant finding is that by using the SGoFincance Trace, one may immediately move to a smaller amount of effects (and FDR) by inspecting other values for the γ parameter. For example, the choice γ = 0.1 reveals 524 true effects. By declaring as true effects the smallest 524 p-values one commits a FDR of about 16%.

In the case of choosing the initially suggested γ value of 0.26, the threshold *p*-value is 0.0516 (0.0393 for the less liberal γ = 0.1). This fact could be taken as surprising at first sight since SGoF is (weakly) controlling the FWER at level 0.05 for each γ value, and the threshold corresponding to γ = 0.26 is above that level. The explanation for this is in the definition of SGoF: for each γ, performs a test at the α level for the null H_0_: E[*K*(γ)] = S*γ, thus controlling the FWER at the given level. In this way, SGoF uses the information contained in the distribution of the whole set of *p*-values (not only in that below 0.05) to reach a conclusion about which ones should be considered as potential true effects. If one is not willing to declare as an effect any *p*-value above α, the obvious modification of SGoF(γ) is simply given by *N*_α_(γ) = min(*K*(γ)−b_α_(γ)+1, *K*(α) ). Clearly, FWER control at the α level remains true when this correction is applied.

The SGoFicance Trace is also useful when the aim is to keep up a given proportion of false positives. In this regard, Figure 3C shows that an FDR of 5% is obtained with SGoF(γ) in the case of γ = 0.83 (threshold *p*-value of 0.003), with 140 genes detected as significant. The difference between this amount and the 160 genes provided by the *q*-value method for the same FDR comes from our more conservative estimation of the proportion of true nulls ([4] worked under the less conservative eπ_0_ = 0.67 rather than our 0.72).

It could be asked what happens to Hedenfalk data's SGoFicance Trace when applied at a different FWER level, more or less conservative than 5%, as for instance 0.01% and 15% respectively. An interesting finding is that the maximum FDR in these two quite extreme cases was never above the maximum FDR of 20% revealed by Figure 3C; namely, a maximum FDR of 17.6% and 19.7% is respectively obtained when the FWER is fixed to be the corresponding α = 0.0001 and α = 0.15. The SGoFicance Trace's maximum FDR showed a similar lack of sensitivity for the FWER in the simulated data above (results not shown). As a conclusion, the default value α = 0.05 could be taken as a good initial choice, with a small impact in the plots of significance. The fact that the SGoF method controls the FWER only in a weak sense is responsible for this robustness.

### Decision guidelines

Some general guidelines can be given for the use of the SGoFicance tool. We can distinguish between two extreme cases. First, the researcher is specially interested in the detection of effects while having information on the corresponding FDR. This can be the case of any exploratory study at the genome or proteome-wide level comparing for example two species. In this case the panel B of the SGoFicance should guide the decision. This panel will immediately tell the researcher the maximum number of true effects that can be detected under SGoF and the FDR that should be assumed in doing so. Moreover, it will inform the researcher about the maximum FDR that should be assumed because a higher one will not translate in more power since SGoF will be unable to find statistical significance for a larger number of true effects. On the other hand, we can think in a second general case where the researcher is interested in minimizing the FDR, as in an association study from which, with limited economic resources, we are going to isolate the detected genes or proteins. In this case the panel C should be the first to be consulted to set the desired FDR and afterwards exploring the corresponding statistically significant number of effects and *p*-value threshold.

As a conclusion, the SGoFicance tool aims to provide solution to the extreme situations above mentioned besides the whole range of intermediate cases where equilibrium between FDR control and power is desired.

## Methods

### Design and Implementation

The algorithms necessary for the computation of the SGoFicance Trace were implemented in a script programmed in the R language [10]. The program asks for an input file that should have an integer number indicating the total number of tests, followed by two columns with pairs of identifiers and *p*-values. The identifier can be a number or a character string. The list of *p*-values does not need to be sorted. This format is the same as for the SGoF program [3]. Next, the program lets the researcher choose the desired α level to control the FWER, and the calculations are performed.

### Availability and Future Directions

The R script for the computation of the SGoFicance is available from the site http://webs.uvigo.es/acraaj/SGoFicance.htm.
